# Methods for Computed Tomography‐Based Glenoid Bone Loss Measurement Show High Interobserver Consistency but Variable Accuracy

**DOI:** 10.1002/ars2.70135

**Published:** 2026-10-28

**Authors:** Ayyappan V. Nair, Aravind Rajan, Kiran Veerendra, Priyamvada M, Yon‐Sik Yoo, Bimal A. Kumar, Shakir Rashid, Adarsh Dalapathi, Tejaswari Jetty, Sarath Reddy Sadda, Prince Shanavas Khan

**Affiliations:** ^1^ Department of Orthopaedics Manipal Hospital, Whitefield Bangalore India; ^2^ Department of Orthopedic Surgery Camp 9 Orthopedic Clinic Hwaseong‐si Gyeonggi‐do Republic of Korea; ^3^ Department of Orthopaedic Surgery N. S. Memorial Institute of Medical Sciences Kollam India; ^4^ Department of Orthopaedics Apollo Adlux Hospital, Angamali Kochi Kerala India

## Abstract

**Purpose:**

To evaluate the reliability of computed tomography (CT)‐based glenoid bone loss calculation methods in shoulder instability by assessing interobserver agreement using 3‐dimensional virtual bone models and to identify the technique that provides the most optimal balance for clinical application.

**Methods:**

Between January 2023 and June 2023, normal shoulder CT scans without evidence of instability or prior surgery were retrieved from institutional archives. Virtual anteroinferior glenoid bone defects of predefined sizes (10%, 15%, 20%, 25%, 30%, 35%, and 40%) were created on a standardized en face glenoid view using CT console‐based segmentation tools, establishing a controlled reference standard. Four senior musculoskeletal radiologists independently measured glenoid bone loss on both 2‐dimensional and 3‐dimensional reconstructions using 4 established techniques (Sugaya, PICO, Hamamoto, and Parada). Interobserver reliability was assessed using the intraclass correlation coefficient (ICC), and agreement with the predefined defect size was evaluated using Bland‐Altman analysis.

**Results:**

The PICO method showed the highest interobserver reliability (ICC = 0.995) but showed systematic overestimation with the largest mean bias (+6.93 mm) and wide limits of agreement, reflecting proportional bias. The Sugaya method showed both high reproducibility (ICC = 0.854) and minimal bias (–1.06 mm), making it the most balanced technique. The Hamamoto method showed good reliability (ICC = 0.952) but a tendency to overestimate (+4.04 mm). The Parada method showed poor reproducibility (ICC = 0.223) despite moderate accuracy (–2.85 mm).

**Conclusions:**

The Sugaya method showed the most favorable combination of accuracy and interobserver reliability for CT‐based glenoid bone loss measurement in a controlled virtual model. The PICO method, despite excellent interobserver consistency, showed systematic overestimation. The Hamamoto method showed acceptable reliability with moderate overestimation, whereas the Parada method showed poor reproducibility.

**Clinical Relevance:**

Reliable glenoid bone loss estimation is vital for surgical planning in shoulder instability. According to our study, the Sugaya method offers the most accurate and reproducible measurements, helping surgeons make better‐informed decisions and avoid inappropriate procedures.

## Graphical abstract

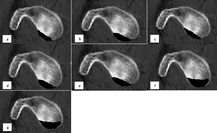

The calculation of glenoid bone loss plays a pivotal role in the treatment of shoulder instability. A surgeon's decision to proceed with a soft tissue procedure or bony augmentation largely depends on the reliable estimation of glenoid bone loss. Over the years, various studies have defined the “critical bone loss,” with a declining trend from 20% to 25% to the currently accepted cutoff of 13.5% in certain patient groups.^1^

Accurate measurement of bone loss is essential to avoid 2 extremes: underestimation, which may lead to recurrent instability and surgical failure, and overestimation, which may result in unnecessary bony augmentation.

Computed tomography (CT) scans are currently recognized as the gold standard for glenoid bone loss calculation, particularly when using the en face view, as they provide high spatial resolution.^2^ Numerous techniques for estimating glenoid bone loss are described in the literature—such as the Sugaya technique,^3^ Hamamoto method,^4^ Parada method,^5^ circle‐line method (CLM), and PICO method.^6^ Each relies on different geometric assumptions, reference points, and estimation strategies.

A major unresolved challenge, however, is interobserver variability. When different radiologists or surgeons apply the same CT‐based method to the same patient, their estimates may vary significantly, potentially altering treatment decisions and outcomes. Thus, the interobserver reliability and accuracy of these methods are critical in evaluating patients with shoulder instability.

The purpose of this study was to evaluate the reliability of CT‐based glenoid bone loss calculation methods in shoulder instability by assessing interobserver agreement using 3‐dimensional (3D) virtual bone models and to identify the technique that provides the most optimal balance for clinical application.

## METHODS

This observational, comparative, image analysis‐based study was conducted between January 2023 and June 2023 at a tertiary care academic center. A normal shoulder CT scan was selected from the institutional picture archiving and communication system database. Inclusion criteria comprised adult patients older than 18 years with no clinical or radiological evidence of shoulder instability, no history of prior shoulder surgery or fracture, and normal glenoid morphology, whereas CT scans showing degenerative changes, prior trauma, motion artifacts, or abnormal glenoid anatomy were excluded. The CT scan was acquired using a multidetector CT scanner with thin‐slice acquisition of 1 mm or less and reconstructed in axial, coronal, sagittal, and en face glenoid views using standard bone reconstruction algorithms. Virtual glenoid bone defects were created on the anteroinferior glenoid rim using CT workstation segmentation and image‐editing software, with the en face glenoid surface area of the intact glenoid serving as the reference standard. Bone defects corresponding to 10%, 15%, 20%, 25%, 30%, 35%, and 40% loss of the inferior glenoid surface area were digitally removed in a stepwise and reproducible manner, as shown in the en face 2‐dimensional (2D) and 3D CT reconstructions (Figures 1 and 2). Because the original glenoid surface area was known and digitally modified, the percentage of bone loss was predefined for each dataset and served as a gold standard reference, allowing objective validation of measurement accuracy. Four senior musculoskeletal radiologists with more than 10 years of experience each evaluated the datasets and were blinded to the predefined defect sizes as well as to each other's measurements. Each observer assessed glenoid bone loss using 4 established measurement techniques:

**FIGURE 1:**
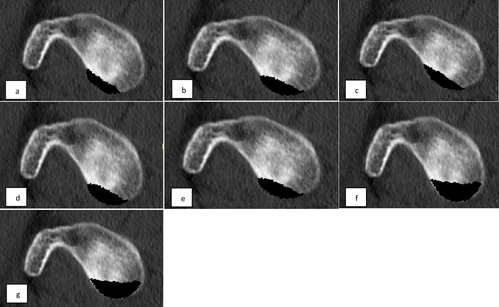
En face 2D CT reconstructions of the glenoid showing digitally created anteroinferior bone defects of predefined sizes (a‐g: 10%, 15%, 20%, 25%, 30%, 35%, and 40%). The original glenoid contour is preserved, allowing standardized comparison across measurement techniques. (2D, two dimensional; CT, computed tomography.)

**FIGURE 2:**
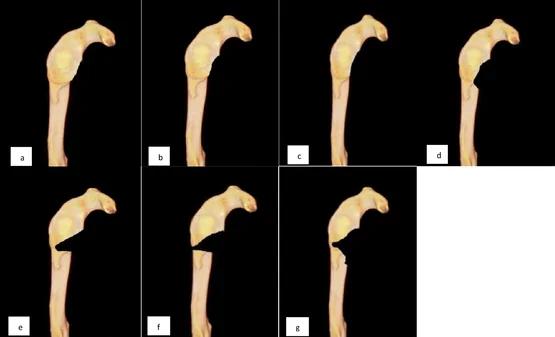
En face 3D CT reconstructions of the glenoid showing digitally created anteroinferior bone defects of predefined sizes (a‐g: 10%, 15%, 20%, 25%, 30%, 35%, and 40%). The original glenoid contour is preserved, allowing standardized comparison across measurement techniques. (3D, three dimensional; CT, computed tomography.)

### Sugaya Method (3D En Face Diameter‐Based Measurement)

This method uses 3D reconstructed CT images of the glenoid in an en face view. A best‐fit circle is applied to the inferior glenoid, representing the presumed intact articular surface. The maximum transverse diameter of the intact glenoid (D) is determined from this circle. The width of the anterior bone defect (d) is then measured as the linear distance missing from the anterior rim. Glenoid bone loss is calculated as a percentage using the formula: (d/D) × 100.^3^

### PICO Method (2D Surface Area‐Based Best‐Fit Circle Method)

In this technique, a 2D en face CT image is used. A best‐fit circle is drawn along the inferior portion of the intact glenoid to estimate the original glenoid surface area. The area of the bone defect is then outlined and calculated. Glenoid bone loss is expressed as the percentage of the defect area relative to the area of the best‐fit circle, providing a surface area‐based assessment rather than a linear measurement.^6^

### Hamamoto Method (Diameter‐Based Technique Using Specialized Measurement Software)

This method involves the use of dedicated imaging software to analyze CT scans in an en face orientation. A best‐fit circle is generated digitally over the inferior glenoid. The software allows precise measurement of the glenoid diameter and quantification of the anterior defect width. Bone loss is calculated as the ratio of defect width to the estimated original glenoid diameter, expressed as a percentage. The use of software improves measurement precision and reproducibility.^4^

### Parada Method (Contralateral Best‐Fit Circle Comparison)

This technique uses the contralateral (unaffected) glenoid as a reference. A best‐fit circle is applied to the intact glenoid to determine the native glenoid size and shape. This circle is then mirrored or scaled to the affected side, allowing estimation of the original glenoid contour. The bone defect is quantified by comparing the deficient anterior rim of the affected glenoid to the reconstructed native circle and expressed as a percentage of bone loss relative to the intact side.^5^

Interobserver reliability was assessed using the intraclass correlation coefficient (ICC) with a 2‐way random‐effects model and absolute agreement, whereas accuracy was evaluated by comparing measured values with predefined defect sizes using Bland‐Altman analysis. Statistical analysis was performed using SPSS software (version 30), and ICC values were interpreted according to established guidelines.

## RESULTS

Among the techniques, the PICO method showed the highest ICC (0.995), indicating excellent reproducibility across observers, followed by Hamamoto (0.952) and Sugaya (0.854), both of which showed good reliability. In contrast, the Parada method showed poor inter‐rater agreement (ICC = 0.223), as summarized in Table 1. PICO, despite its exceptional ICC, exhibited the largest mean bias (+6.93 mm) and the widest limits of agreement, indicating systematic overestimation that became more pronounced with larger defect sizes, which is a clear example of proportional bias, where the method was precise but inaccurate. The Sugaya technique, in contrast, showed minimal mean bias (–1.06 mm) with moderate limits of agreement, reflecting both accuracy and reproducibility. The Hamamoto method showed a moderate bias (+4.04 mm) with narrower limits than PICO, suggesting acceptable performance but with a tendency to overestimate. The Parada method, while displaying poor reproducibility, had a mean bias of –2.85 mm, suggesting fair accuracy under controlled conditions, as summarized in Table 2. Overall, the Sugaya technique emerged as the most balanced method, combining accuracy with reliability; Hamamoto performed acceptably although with systematic overestimation; PICO, although highly consistent, proved systematically inaccurate; and Parada lacked sufficient reproducibility to be clinically dependable.

**TABLE 1:** ICC Values

**Method**	**ICC**
Hamamoto	0.952
Pico	0.995
Parada	0.223
Sugaya	0.854

ICC, intraclass correlation coefficient.

**TABLE 2:** Bland‐Altman Analysis Output

**Method**	**Mean Difference**
Hamamoto vs std.	4.0411
Pico vs std.	6.9257
Parada vs std.	−2.8475
Sugaya vs std.	−1.0629

std, standard.

## DISCUSSION

In this controlled virtual CT study, we found that CT‐based methods for glenoid bone loss measurement show marked differences between interobserver reproducibility and true measurement accuracy, with the Sugaya method providing the most balanced performance. This significant variation in the accuracy and interobserver reliability among different CT‐based methods for glenoid bone loss estimation may directly influence surgical decision‐making and ultimately impact patient outcomes after shoulder instability correction procedures. Glenoid bone loss occurs in up to 22% of patients after an initial shoulder dislocation.^7^ With recurrent episodes, the extent of bone loss increases, progressively reducing the glenoid‐humeral contact area and perpetuating instability.^8^ A systematic review by Min et al. showed that glenoid bone loss of ≥16.0% significantly increases the risk of recurrence after arthroscopic Bankart repair alone.^9^ These findings underscore the importance of accurate bone loss measurement as a critical determinant in surgical planning.

Previous literature has highlighted variability in the accuracy and reproducibility of available measurement methods. Obana et al. reported good reproducibility of the best‐fit circle technique on 3D CT among both orthopaedic surgeons and radiologists,^10^ whereas Green et al., in a systematic review, concluded that 3D CT with surface area measurement is the most reliable and accessible approach at present, although none of the CT‐based techniques can be considered ideal.^11^

However, the 3D CT glenoid bone loss calculations can be imperfect because of the variations in scapular tilt and 3D en face view selection during the study.^12^ In the present study, virtual glenoid bone cuts were created on normal CT scans in 2D and 3D formats to eliminate potential bias in reporting caused by en face view selection, which is one of the key strengths of this work. Both interobserver variability and the reliability of the different techniques were subsequently analyzed.

The results showed that ICC is highest for PICO method (0.995). The PICO method is a surface area‐based technique for glenoid bone loss calculation that uses the multiplanar reformation shifting technique in 2D CT.^13^ However, despite showing the highest agreement among radiologists, it also had the largest mean bias when analyzed with Bland‐Altman plots, making it the least accurate method overall. Specifically, the PICO method showed the highest inter‐rater reliability (ICC = 0.995), indicating excellent reproducibility. However, it exhibited the highest mean bias (+6.93 mm) and the widest limits of agreement, reflecting poor validity. This discrepancy highlights a key statistical nuance: ICC measures precision (consistency among raters), not accuracy. A method can have high ICC if all raters consistently make the same error. In this case, the PICO method systematically overestimated bone loss, likely because of proportional bias, where the error increased with larger defect sizes. Thus, the PICO method was precise but not accurate. Similar findings were reported by Tennent et al., who observed that although the PICO method showed the highest ICC, it was less accurate at lower glenoid bone loss (~13.8%) but performed reasonably well at higher defect sizes.^6^

The Sugaya method of CT glenoid bone loss uses 3D en face view for CT bone loss calculation, as described by Sugaya et al.^3^ This technique showed excellent reproducibility and minimal mean bias (−1.06), making it the most favorable technique in our study. Chen et al. in their study investigated a comparison card to evaluate the glenoid bone defect compared against the Sugaya method and showed reasonable interobserver reliability with excellent accuracy.^14^

The Parada technique, which is based on contralateral glenoid measurement for creating the best‐fit circle to calculate glenoid bone loss, showed poor interobserver correlation (ICC = 0.223). However, the Bland‐Altman analysis shows better mean bias (−2.847), proving better accuracy. This suggests that although accuracy may be acceptable under standardized conditions, variability between users limits its clinical utility. Based on the best‐fit circle and CLM, the Parada technique has been shown to provide good accuracy, particularly for bone loss calculations of less than 25% of the glenoid.^5^

The PICO method, which uses the circle of best fit of the contralateral side on 2D CT scans, showed low accuracy despite showing good interobserver reliability. Studies by Tennet et al. showed good accuracy (97%) and a positive predictive value (100%), especially at higher glenoid bone loss (17.6%).^6^ Similarly, Zappia et al., in a multicentric study evaluating imaging modalities for interobserver agreement in glenoid bone loss, found PICO to be the most reliable method.^15^ However, its consistent overestimation and poor Bland‐Altman agreement in our data suggest that it should be used with caution or recalibrated for clinical application.

Hamamoto et al. using a specialized software program devised a technique involving both diameter‐ and surface area‐based methods. They showed that the diameter‐based technique tended to exaggerate bone loss, particularly around 20% glenoid bone loss.^4^ In our study, Hamamoto method showed good inter‐rater reliability (ICC = 0.952) but a moderate mean bias (+4.04 mm), suggesting systematic overestimation. These findings are consistent with prior literature, which has reported similar tendencies of the diameter‐based approach to exaggerate bone loss. Although our test radiologists also showed high mean bias, reflecting poor accuracy, the method still showed good interobserver reliability.

A key finding of this study is the distinction between reproducibility and accuracy. Although high interobserver agreement is often interpreted as methodological superiority, our results show that consistency alone does not ensure validity. The PICO method exemplifies this limitation, showing excellent reproducibility yet systematic overestimation, emphasizing that both accuracy and reliability must be jointly considered when selecting a clinically meaningful measurement technique.

Traditionally, 3D CT scans have been considered the preferred modality for quantifying glenoid bone loss.^2^ However, magnetic resonance imaging is gradually gaining popularity, particularly 3D magnetic resonance imaging techniques, which have shown equivalent reliability and accuracy to 3D CT methods while avoiding the risk of ionizing radiation exposure.^16^ Nonetheless, availability and cost remain substantial limiting factors.^17^

From a methodological standpoint, this study is strengthened by the use of virtual bone loss models with predefined defect sizes, which served as a gold standard for evaluation. This approach allows for a more objective assessment than studies compared with studies based on clinical CTs where the true bone loss is unknown. Furthermore, the use of 4 blinded observers across 4 methods enhances the generalizability of our findings.

### Limitations

This study is not without limitations. First, although virtual models ensure consistency, they may not fully capture the full spectrum of clinical variability encountered in practice, such as patient positioning, CT artifacts, or irregular defect morphology. Second, our observer pool consisted only of radiologists, and results may differ if orthopaedic surgeons were included as raters. Third, although the 7 models represented a spectrum of defect severities, the sample size remains relatively limited. Additionally, because the defects were digitally created, the residual contour of the native glenoid anatomy may have remained partially perceptible, potentially influencing observer perception and introducing measurement bias compared with naturally occurring bone loss. Finally, sex‐based analysis was not performed because the study used a single anonymized normal CT dataset with digitally created defects, precluding assessment of sex‐specific anatomical variability.

## CONCLUSIONS

The Sugaya method showed the most favorable combination of accuracy and interobserver reliability for CT‐based glenoid bone loss measurement in a controlled virtual model. The PICO method, despite excellent interobserver consistency, showed systematic overestimation. The Hamamoto method showed acceptable reliability with moderate overestimation, whereas the Parada method showed poor reproducibility.

## DISCLOSURES

The authors (A.V.N., A.R., K.V., P.M., Y‐S.Y., B.A.K., S.R., A.D., T.J., S.R.S., P.S.K.) declare that they have no known competing financial interests or personal relationships that could have appeared to influence the work reported in this article.
